# Reply: Improved safety and effectiveness of imaging predicted for MR mammography

**DOI:** 10.1038/sj.bjc.6601443

**Published:** 2004-01-06

**Authors:** P J Kneeshaw, L W Turnbull, P J Drew

**Affiliations:** 1 Academic Surgical Unit, Castle Hill Hospital, Castle Road, Cottingham, HU16 5JQ, UK; 2 The Centre for Magnetic Resonance Investigations, Hull Royal Infirmary, Hull, UK

**Sir**,

We read with interest the comments regarding a possible relationship between female carriers of ataxia telangiectasia (AT) with susceptibility to low-dose ionising radiation and the induction of breast cancers in women of screening age.

Although MR mammography does not use ionising radiation, its use in screening of women at a high risk of breast cancer is currently being evaluated, both because of the inherent high sensitivity of the technique, especially in dense pre-menopausal breast tissue and the safety issues particularly in Li-Fraumeni syndrome. The UK Magnetic Resonance Imaging for Breast Screening study (MARIBS) is a multicentre ongoing trial comparing the efficacy of X-ray mammography and MRI as a method for screening genetically high-risk women aged 35–50 years. This trial will close in 2005. Results from Kuhl *et al* (2003) and Kriege *et al* (2003) presented recently at the American Society of Clinical Oncology, relating to similar trials again of genetically high-risk subjects have indicated promising results. The authors are unaware of any current trials comparing imaging modalities for screening the 50–64-year age group, or indeed if such a trial is contemplated. The results of such a comparison including health economic issues would need to be very carefully evaluated before embarking on a long-term follow-up trial to elucidate the risks of screening using techniques dependent on ionising radiation.
